# Infection through the Air

**Published:** 1898-02

**Authors:** 


### Infection Through the Air.

C. Fluegge reports (*Ztft. j. Hyg. u. Infect.*, Vol. 25, No. 1), the result of much research in regard to the transmission of germs through the air. Dust from matter containing bacteria always consists partially of fragments so minute that they float in the air of a quiet room for more than four hours, and the slightest current of air will waft them about. He also states that tiny drops of moisture containing bacteria pass into the air even in closed rooms from the ordinary manipulations and float for five hours, and are transported by a current no stronger than 0.07 m. m. for horizontal draft, or 0;1 m. m. an upward current. The dissemination of these transportable drops is of great importance in regard to the secretions of the mouth and nose, especially in case of tuberculosis, influenza, diphtheria, etc. He found by repeated tests that plates of agar become covered with colonies of bacteria at a distance of several yards, from persons speaking loudly and energettically. Coughing produced a still more rapid and abundant colonization, while the plates showed no growth when the person spoke low and quietly. He does not consider dry particles floating as dry dust in the air a serious menace for the infection of wounds during operations, as the staphylococcus aureus and albus in these conditions are not sufficiently virulent to colonize agar plates ; all his experiments in this line were negative. But he emphasizes the danger to be apprehended from the drops of moisture disseminated from the secretions of the mouth and nose of the persons present at the operation, as they speak, sneeze or cough. The pyogenic bacteria are frequently found in the mouth of perfectly healthy persons, and decayed teeth are a mine of all sorts of bacteria. These microbes have not been attenuated by drying, but are moist and often extremely virulent. Distance from the operating table does not prevent the germ-laden drops of moisture from reaching the patient or some following case or alighting on the instruments. The patient himself may infect the field by heavy breathing, etc. Mikulicz's "Transcendental Surgery," described page 201 of the *Journal* (gloves and mouth screens), was inspired principally by these experiments of Fluegge, with which he was familiar.—*Cbl. f. Chir.*, October 2.

